# ADAR3 expression is an independent prognostic factor in lower-grade diffuse gliomas and positively correlated with the editing level of GRIA2^Q607R^

**DOI:** 10.1186/s12935-018-0695-8

**Published:** 2018-12-03

**Authors:** Ying Zhang, Kuanyu Wang, Zheng Zhao, Si Sun, Kenan Zhang, Ruoyu Huang, Fan Zeng, Huimin Hu

**Affiliations:** 1 Department of Molecular Neuropathology, Beijing Neurosurgical Institute, Capital Medical University, Beijing, China; 2 Chinese Glioma Cooperative Group (CGCG), Beijing, China; 3 Department of Neurosurgery, Beijing Tiantan Hospital, Capital Medical University, Beijing, China; 4 Department of Radiotherapy, Beijing Tiantan Hospital, Capital Medical University, Beijing, China

**Keywords:** ADAR3, A-to-I editing, Prognosis, GRIA2^Q607R^, Lower-grade glioma

## Abstract

**Background:**

RNA editing by adenosine deaminases acting on RNA (ADARs) converts adenosines to inosines (A-to-I) in RNA, that alters gene expression and generates protein diversity. Dysregulation of A-to-I editing has been found associated with a number of nervous system diseases. However, the role of ADAR3, a brain specific high expression adenosine deaminase, in gliomas has rarely been investigated. In this study we illuminated the clinical significance and molecular features of ADAR3 in patients with glioma.

**Methods:**

309 glioma samples from Chinese Glioma Genome Atlas were enrolled into this study. In validation sets, 601 glioma samples in TCGA, 410 glioma samples in REMBRANDT and 258 glioma samples in GSE16011 were obtained. Relationships between *ADAR3* expression and prognosis-related genomic alteration, outcome and gene ontology analysis were investigated. Moreover, the characteristic of GRIA2^Q607R^ editing in gliomas has been investigated. Graphpad Prism 5.0, SPSS 16.0 and R language were used to perform statistical analysis and graphical work.

**Results:**

*ADAR3* expression was down regulated along with glioma grade progression in CGGA dataset. *ADAR3* was characteristically highly expressed in neural subtype and *IDH1/2* mutant preference. Moreover, high expression of *ADAR3* predicted a better prognosis in lower-grade glioma (LGG) patients and multivariate analysis suggested *ADAR3* expression was an independent prognostic indicator. The results of the three other validation datasets showed similar findings. Bioinformatics analyses suggested that ADAR3 may play a role in the malignant transformation of glioma cells by affecting cell proliferation, angiogenesis or cell adhesion. Furthermore, the editing level of GRIA2^Q607R^ was significantly correlated with ADAR3 expression.

**Conclusions:**

Our study demonstrated the clinical and molecular characterization of ADAR3 in glioma development and progression. ADAR3 expression was negatively associated with tumor malignant in the overall glioma patients. And it was a favorable independent prognostic indicator of LGG patients. ADAR3 appeared to act as a tumor suppressor in glioma cells. Therefore, ADAR3 represented a potential therapeutic target and useful prognostic factor for glioma patients.

**Electronic supplementary material:**

The online version of this article (10.1186/s12935-018-0695-8) contains supplementary material, which is available to authorized users.

## Background

Gliomas are the most common primary intracranial tumor, representing 81% of malignant brain tumors [1]. According to 2007 World Health Organization (WHO) classification criteria, gliomas can be divided into four grades based on the degree of malignancy (WHO Grade I-IV). WHO Grade I apply to lesions with low proliferative potential and the possibility of cure following surgical resection alone. WHO Grade II and III are called lower-grade gliomas (LGG) [2]. Because of they are generally infiltrative in nature, surgical resection is not enough, and patients may need adjuvant radiation and/or chemotherapy [3]. WHO grade IV (reserved for glioblastoma) is the most malignant form of glioma, which has a 5-year relative survival of ~ 5% [1]. Studies over the past two decades have clarified several genetic alterations in gliomas, such as mutations in *IDH1/2*, *TP53* and *ATRX*, *TERT* promoter mutation, *MGMT* promoter methylation and 1p/19q co-deletion, etc. Some of them contribute to glioma classification, prognosis or guidance in therapeutic decisions. In the 2016 WHO classification of central nervous system (CNS) tumors, classification of diffuse gliomas (WHO Grade II-IV) has fundamentally changed: for the first time, a large subset of these tumors is now defined based on *IDH1* or *IDH2* mutation and co-deletion of chromosomal arms 1p and 19q [4]. This breaks with the principle of diagnosis based entirely on phenotypic by incorporating genotypic parameters into the classification of CNS tumor entities [4]. The exploit of novel and reliable biomarkers for the prediction of gliomas may further help to elucidate the molecular mechanism of glioma development and progression.

RNA editing is one of the posttranscriptional mechanisms that precisely alters RNA sequences, thus regulates gene expression and generates structurally or functionally different isoforms of proteins. The most predominant pattern of RNA editing converts adenosine to inosine (A-to-I) in coding and non-coding RNA sequences, which is mediated by ADAR enzymes [5]. A-to-I editing is most abundant in the CNS and is critical for maintaining proper neuronal function [6]. Targets of this type of RNA editing are transcripts encoding proteins involved in neurotransmitter receptors and voltage-gated ion channels, including α-amino-3-hydroxy-5-methyl-4-isoxazole-propionic acid (AMPA) [6], potassium channel Kv1.1 (KCNA1) [7], G protein-coupled serotonin receptor 5-HT_2C_R (5-hydroxytryptamine receptor subtype 2C) [8] and the α3 subunit of GABA_A_ (γ-aminobutyric acid type A) receptor (GABRA3) [9]. Changes in the A-to-I editing have been associated with a number of human diseases, such as amyotrophic lateral sclerosis (ALS), transient forebrain ischemia, epilepsy, metastatic melanoma, glioblastoma (GBM, WHO grade IV) and hepatocellular carcinoma (HCC) [6, 10, 11].

In human, three ADAR family members (ADAR1, ADAR2 and ADAR3) and two ADAD (adenosine deaminase domain-containing) proteins (ADAD1 and ADAD2) have been identified. They all contain at least one dsRNA binding domain (dsRBD) and a conserved C-terminal deaminase domain, whereas ADAR3 contains a unique Arg-rich ssRNA binding domain (R domain) at its N-terminus [12]. Unlike the wide expression of ADAR1 and ADAR2, ADAR3 expression is restricted to the brain [13]. Moreover, ADAR3 is not catalytically active and is thought to act as a competitive inhibitor of ADAR1 and ADAR2 in the brain [13]. Recently, ADAR3 was proved to directly compete with ADAR2 for binding to *GRIA2* pre-mRNA, inhibiting RNA editing at the Q607R editing site of *GRIA2* in GBM cell line [14]. This editing site is almost 100% edited in mammalian brain and controls the calcium permeability of AMPA receptor channels, which are involved in fast excitatory synaptic transmission [15, 16]. These studies suggest that ADAR3 may be associated with the tumorigenesis and progression of glioma. However, the clinical significance and molecular features of glioma with ADAR3 expression remain elusive.

In this study, we evaluated the expression pattern, prognostic significance and potential biological association of ADAR3 in patients with glioma. We collected *ADAR3* mRNA expression and clinical information in 1578 glioma samples from four independent datasets. Meanwhile, the expression pattern of *ADAR3* in different types of gliomas was evaluated by *t* test and one-way ANOVA test. In addition, the overall survival of glioma patients was assessed based on *ADAR3* expression level and the prognostic value of ADAR3 in glioma was tested using Cox regression analysis. Furthermore, the bioinformatics analyses were applied to predict the biological process of ADAR3 in gliomas. Finally, the relationship of GRIA2^Q607R^ editing and ADAR3 expression has been analyzed based on CGGA RNAseq dataset. These results suggested that ADAR3 was a novel independent prognostic indicator of LGG patients and appear to act as a tumor suppressor in glioma cells.

## Methods

### Patients and samples

309 specimens of WHO grade II–IV from Chinese Glioma Genome Atlas (CGGA) were included in our study. This study was approved by the Institutional Review Boards of Beijing Tiantan Hospital (Beijing, China). The written informed consents were obtained from all the participants enrolled in the study. All experiment methods were performed in accordance with the relevant guidelines and regulations. The establishment and management of our CGGA databank have been reported previously [17].

### ADAR3 expression analysis in datasets

The CGGA RNA sequencing (RNAseq) database (http://www.cgga.org.cn) with 309 glioma samples (104 grade II, 67 grade III, and 138 grade IV samples) were obtained as the discovery set. In validation sets, 601 glioma samples (211 grade II, 236 grade III, and 154 grade IV samples) in The Cancer Genome Atlas (TCGA) RNAseq database (http://cancergenome.nih.gov), 410 glioma samples (99 grade II, 84 grade III, and 227 grade IV samples) in The Repository for Molecular Brain Neoplasia Data (REMBRANDT, http://caintegrator-info.nci.nih.gov/REMBRANDT), and 258 glioma samples (23 grade II, 84 grade III, and 151 grade IV samples) in GSE16011 microarray database (https://www.ncbi.nlm.nih.gov/geo/) were obtained. The raw data were centralized and standardized through the scale function in R language before analysis. In the four datasets, only samples classified WHO grade II-IV were included for survival and grade expression pattern analysis.

### Characterization of A-to-I RNA editing of GRIA2^Q607R^

RNAseq library preparation, sequencing and RNAseq data analysis were processed as our previous research [18]. Then we obtained the RNAseq BAM files of 309 glioma samples in the CGGA RNAseq database. On the basis of the RNAseq reads mapped to the human reference genome (hg19), the editing level at Q607R site of GRIA2 in a given sample was calculated as the number of edited reads G divided by the total number of reads A + G (G/A + G), and the total number of reads A + G less than 30 were excluded.

### Statistical analysis

Student’s *t*-test and one-way ANOVA test were used to test the significance of differences between patients with different grades of gliomas by R language. Overall survival time (OS) was calculated from the date of histological diagnosis until death or the last follow-up. Kaplan–Meier survival analysis and the log-rank test were used to assess the statistical significance between stratified survival groups. Half of the patients who had relatively higher ADAR3 expression were defined as high expression group, while the other half part of patients were considered as lower expression group. Univariate and multivariate Cox regression analysis including gender, age at diagnosis, WHO grade, *MGMT* promoter methylation, *IDH1/2* mutation status, radiotherapy and chemotherapy were used to assess prognostic value of ADAR3 in glioma by using SPSS 16.0 (Armonk, NY, USA). A *P* value less than 0.05 was considered to be statistical significant.

The Pearson correlation analysis was performed by R language to calculate the correlation between ADAR3 and other genes in CGGA RNAseq datasets. GO and KEGG pathway analysis were performed using the online Database for Annotation, Visualization and Integrated Discovery (DAVID, http://david.abcc.ncifcrf.gov).

The mRNA expression profile of glioma samples from the CGGA mRNA sequencing was analyzed by gene set enrichment analysis (GSEA, http://www.broad.mit.edu/gsea). For GSEA, the expression level of ADAR3 was divided into low- or high-expression group by a criterion of whether the expression level was greater than the median value.

## Results

### *ADAR3* expression is down regulated along with glioma grade progression and shows a subtype preference

**Fig. 1:**
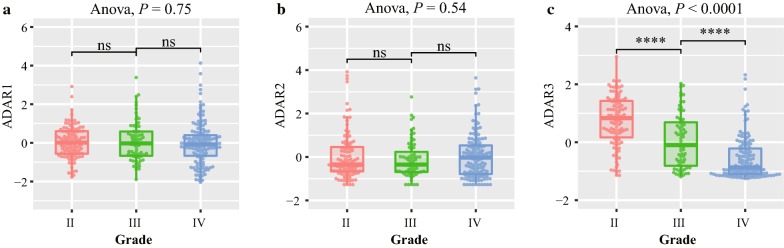
Expression analysis of ADAR1, ADAR2 and ADAR3 in diffuse gliomas. ADAR1 (**a**), ADAR2 (**b**) and ADAR3 (**c**) expression patterns across WHO grade II-IV in CGGA RNA sequencing dataset. ****P < 0.0001; ns, indicates no significant

To get an overview of adenosine deaminase domain containing proteins status in gliomas, we firstly investigated the mRNA expression of these proteins in patients. *ADAD1* and *ADAD2* were discarded for their rarely expression in CGGA mRNA sequencing database (CGGA RNAseq). This may be due to their specifically expression in the testes and necessary for spermatogenesis [19]. Then we compared the mRNA expression of *ADAR1*, *ADAR2* and *ADAR3* in patients with different WHO grades (II, III and IV) based CGGA RNAseq. Among them, only *ADAR3* expression showed a significant negative correlation with tumor grade (*P* < 0.0001, Fig. 1c). And the expression of *ADAR3* also decreased along with grade progression in TCGA RNA sequencing (TCGA RNAseq), GSE16011 and REMBRANDT microarray databases (*P* < 0.0001, Additional file 1: Figure S1C, F and I). Although the expression of *ADAR1* and *ADAR2* was lowest in glioblastoma (GBM, WHO grade IV) of TCGA RNAseq (Additional file 1: Figure S1A, B), the same results did not validated in GSE16011 and REMBRANDT microarray databases (Additional file 1: Figure S1D, E, G, H). These results indicated that *ADAR3* expression associated with malignant progression of glioma.

**Fig. 2:**
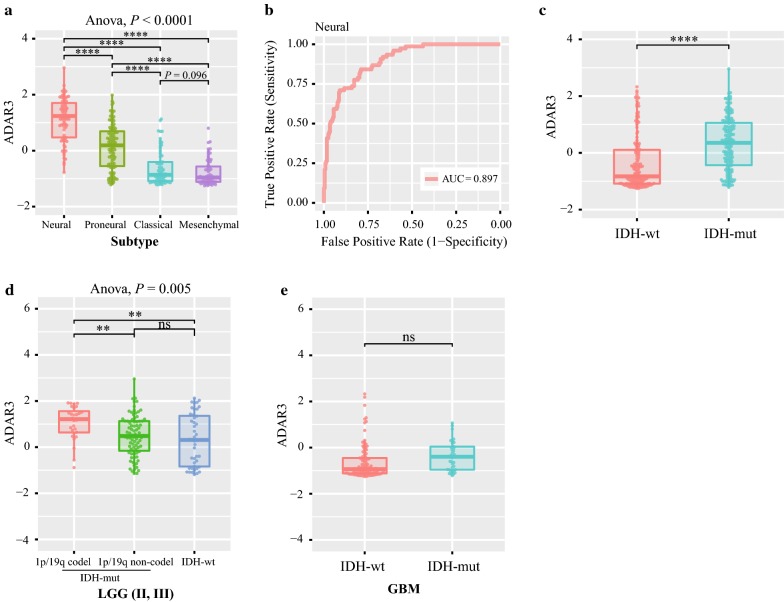
*ADAR3* expression in stratified patients based on CGGA dataset. **a** *ADAR3* expression among different subtypes of patients. **b** The receiver operator characteristic (ROC) curve to predict the neural subtype according to *ADAR3* expression. **c** *ADAR3* expression between IDH-wt and IDH-mut patients. **d** *ADAR3* expression between LGG IDH-mut and IDH-wt patients. **e** *ADAR3* expression between GBM IDH-wt and IDH-mut patients. ****P < 0.0001; **P < 0.01; ns, indicates no significant

To analyze the molecular relevance of ADAR3 in glioma, we investigated the characteristic of *ADAR3* expression in different molecular subtypes established by TCGA network [20]. The neural subtype had the highest *ADAR3* expression, followed by proneural subtype in CGGA, TCGA, GSE16011 and REMBRANDT datasets. Although the differences between classical subtype and mesenchymal subtype is inconsistent in four datasets, the expression of *ADAR3* was still the lowest in these two subtypes (Fig. 2a, Additional file 2: Figure S2A–C). For the further investigation, the gliomas were divided into two groups, neural subtype and non-neural subtype. Receiver operating characteristic (ROC) analysis for ADAR3 mRNA in prediction of neural were performed in four datasets, we found that ADAR3 had a high efficiency to predict neural subtype (Fig. 2b, Additional file 2: Figure S2D–F). This results indicated that ADAR3 could serve as a biomarker for neural subtype.

Then we investigated the correlation between ADAR3 mRNA expression level and IDH1 or/and IDH2 (IDH) mutation, which is a canonical indicator of glioma [21]. The patients with mutant IDH (IDH-mut) showed much stronger expression of ADAR3 than those with wildtype IDH (IDH-wt) in CGGA and TCGA datasets (Fig. 2c, Additional file 2: Figure S2G). We further detected its expression in diffuse gliomas patients based on grade, IDH and 1p/19q status. *ADAR3* expression was highest in LGG IDH-mut and 1p/19q codeleted stratified patients (Fig. 2d, Additional file 2: Figure S2H). The difference between GBM IDH-mut and GBM IDH-wt is not significant, but the mean value of ADAR3 in GBM IDH-mut still showed an upward trend compared with that of GBM IDH-wt (Fig. 2e, Additional file 2: Figure S2I). These results indicated that ADAR3 had a IDH mutant preference.

Moreover, we conducted an overview of *ADAR3* expression with several genetic alterations which are related with progression of glioma (Fig. 4a, Table 1). Consistent with the above results, lower WHO grade, neural subtype and IDH mutants are enriched in higher *ADAR3* expression (*P* < 0.0001). Meanwhile, deletion of 1p or/and 19q, confirming favorable prognostic indicators [22], aggregated in gliomas with higher *ADAR3* expression (*P* < 0.0001). These results indicated that higher *ADAR3* expression may be an optimistic factor for patients with glioma.

**Table 1:** Characteristics of glioma patients in low and high ADAR3 expression group in CGGA dataset

Variable	Total (n = 309)	High ADAR3 expression (n = 154)	Low ADAR3 expression (n = 155)	*P* value
Age				
< 45	178	104	74	*0.0005*
≥ 45	131	50	81	
Gender				
Male	115	57	58	1
Female	194	97	97	
Grade				
II	104	86	18	< *0.0001*
III	67	35	32	
IV	138	33	105	
KPS				
< 80	58	42	16	*0.0006*
≥ 80	108	46	62	
NA	143	67	76	
Subtype				
Neural	76	71	5	< *0.0001*
Proneural	99	61	38	
Classical	69	14	55	
Mesenchymal	65	8	57	
IDH mutation				
Mutant	155	108	47	< *0.0001*
Wildtype	154	46	108	
MGMT				
Unmethylated	111	48	63	*0.0462*
Methylated	136	67	69	
NA	62	39	23	
1p/19q deletion				
No	220	102	118	< *0.0001*
Yes	36	31	5	
NA	53	21	32	
TERT				
No	158	79	79	0.6426
Yes	91	43	48	
NA	60	33	27	

### ADAR3 is an independent prognostic indicator for LGG patients

**Fig. 3:**
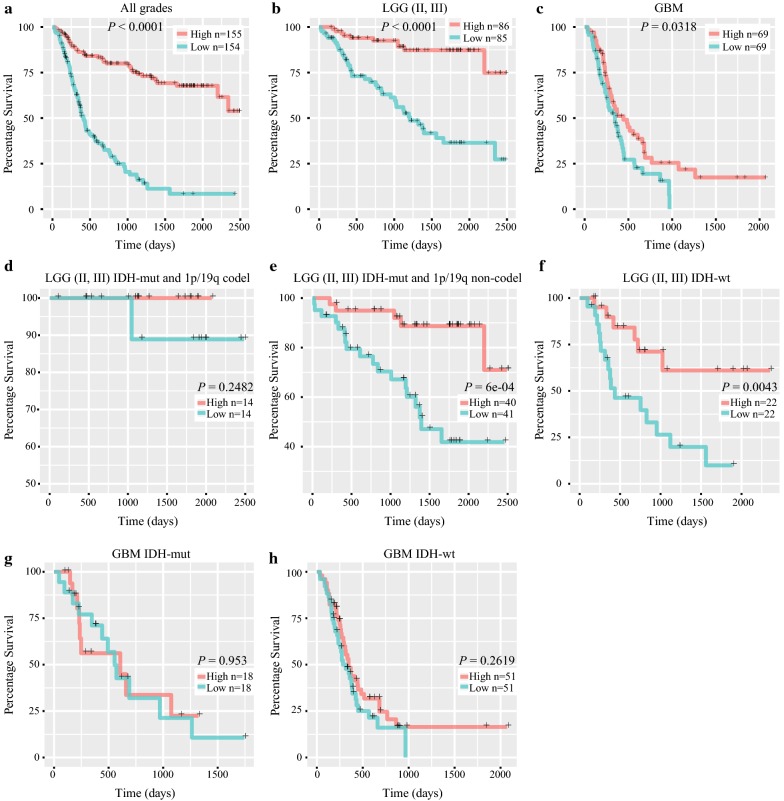
Survival analysis in patients stratified by grade, IDH and 1p/19q status based on ADAR3 expression in CGGA RNAseq dataset. **a**–**c** Comparison of the OS between high and low *ADAR3* expression group in all grades, LGG and GBM patients. **d**–**f** Kaplan–Meier analysis of *ADAR3* expression in LGG stratified by IDH and 1p/19q status. **g**, **h** Kaplan–Meier analysis of *ADAR3* expression in GBM IDH-mut and IDH-wt patients. OS, overall survival; LGG, lower-grade glioma; GBM, glioblastoma

To evaluate the prognostic value of *ADAR3* expression, Kaplan–Meier survival analyses were performed in CGGA RNAseq, TCGA RNAseq, GSE16011 and REMBRANDT datasets, using the median *ADAR3* expression as a cut-off. When taking all grades of patients into account, half of the patients who had relatively higher *ADAR3* expression had a significantly longer survival time than those had lower *ADAR3* expression in four datasets (Log-rank test, all *P* < 0.0001, Fig. 3a, Additional file 3: Figure S3A). Moreover, the prognostic value of *ADAR3* expression was also analyzed in patients with lower-grade glioma (LGG, WHO grade II-III) and GBM, respectively. There was a significant correlation between low expression of *ADAR3* and decreased overall survival rate in LGG patients (Log-rank test, all *P* < 0.05, Fig. 3b, Additional file 3: Figure S3B). Except to CGGA RNAseq dataset (Log-rank test, *P* = 0.0318, Fig. 3c), we failed to identify such significant differences in GBM patients (Log-rank test, all *P* > 0.05, Additional file 3: Figure S3C). Moreover, the similar trend was observed in LGG IDH-mut and 1p/19q non-codeleted and LGG IDH-wt patients (*P* = 6e − 04, *P* = 0.0043, Fig. 3e, f). CDKN2A loss is associated with shorter overall survival in LGG IDH-mut and 1p/19q non-codeleted patients [23]. We further compared ADAR3 expression in this group, the patients with CDKN2A loss showed much lower expression of ADAR3 than those with intact CDKN2A in TCGA dataset (*P* = 0.0247, Additional file 4: Figure S4). Although no significant difference was found in LGG IDH-mut and 1p/19q codeleted patients, it probably due to the small sample size (*P* = 0.2482, Fig. 3d). There was no significant difference in GBM IDH-mut and GBM IDH-wt patients (*P* = 0.953, *P* = 0.2619, Fig. 3g, h). Our results suggest that *ADAR3* expression was at least a prognostic indicator in patients with lower-grade diffuse gliomas.

Furthermore, univariate and multivariate analyses were utilized to evaluate the independent value of *ADAR3* expression and other clinic pathological variables predicting overall survival (OS) in glioma patients. As shown in Table 2, factors including age at diagnosis, WHO Grade, *ADAR3* expression, *MGMT* promoter methylation, *IDH1/2* mutation status and radiotherapy were significantly associated with the OS of glioma patients. Multivariate Cox regression analysis indicated that *ADAR3* expression was an independent prognostic indicator for the OS of glioma patients in CGGA dataset (Table 2, HR, 0.571; 95% CI 0.430–0.760; *P *< 0.001). Moreover, we performed univariate and multivariate analyses in patients with LGG or GBM separately. As shown in Table 2, *ADAR3* expression was an independent prognostic indicator for the OS of LGG patients in CGGA dataset (Table 3, HR, 0.419; 95% CI, 0.289–0.608; *P *< 0.001), but not for the OS of GBM patients (Table 3, HR, 0.804; 95% CI, 0.556–1.163; *P* = 0.247). These results indicate that *ADAR3* expression is an independent prognostic factor in patients with LGG.

**Table 2:** Clinic pathologic factors associated with OS in the Cox hazard regression analysis for glioma patients from the CGGA dataset

Variable	Univariate Cox Regression			Multivariate Cox Regression		
	HR	95% CI	*P* value	HR	95% CI	*P* value
Gender	1.187	0.841–1.675	0.330			
Age at diagnosis	1.038	1.022–1.053	< *0.001*	0.998	0.979–1.018	0.875
WHO Grade	3.469	2.709–4.443	< *0.001*	1.926	1.370–2.709	< *0.001*
*ADAR3* expression	0.356	0.284–0.447	<* 0.001*	0.571	0.430–0.760	< *0.001*
*MGMT* promoter methylation	0.529	0.374–0.750	<* 0.001*	0.665	0.444–0.997	*0.048*
*IDH1/2* Mutation status	0.229	0.159–0.331	< *0.001*	0.637	0.370–1.097	0.104
Radiotherapy	0.421	0.291–0.611	<* 0.001*	0.409	0.276–0.606	< *0.001*
Chemotherapy	1.386	0.969–1.983	0.074			

*P* value < 0.05 was considered statistically significant

CI, confidence interval; HR, hazard ratio; IDH1/2, isocitrate dehydrogenase 1/2; MGMT, O6-methylguanine-DNA methyltransferase

**Table 3:** Clinic pathologic factors associated with OS in the Cox hazard regression analysis for LGG and GBM patients from the CGGA dataset

Variable	Lower-grade glioma				Glioblastoma			
	Univariate	Multivariate			Univariate	Multivariate		
	*P* value	HR	95% CI	*P* value	*P* value	HR	95% CI	*P* value
Gender	0.783				0.355			
Age at diagnosis	< *0.001*	1.023	0.992–1.054	0.143	0.569			
*ADAR3* expression	< *0.001*	0.419	0.289–0.608	< *0.001*	*0.035*	0.804	0.556–1.163	0.247
*MGMT* promoter methylation	0.225				*0.010*	0.551	0.342–0.886	*0.014*
*IDH1/2* mutation status	< *0.001*	0.595	0.290–1.221	0.157	0.089			
Radiotherapy	0.056				< *0.001*	0.447	0.273–0.733	*0.001*
Chemotherapy	*0.002*	1.397	0.705–2.770	0.338	< *0.001*	0.441	0.271–0.717	< *0.001*

*P* value < 0.05 was considered statistically significant

CI, confidence interval; HR, hazard ratio; IDH1/2, isocitrate dehydrogenase 1/2; MGMT, O6-methylguanine-DNA methyltransferase

### ADAR3 related biological process

**Fig. 4:**
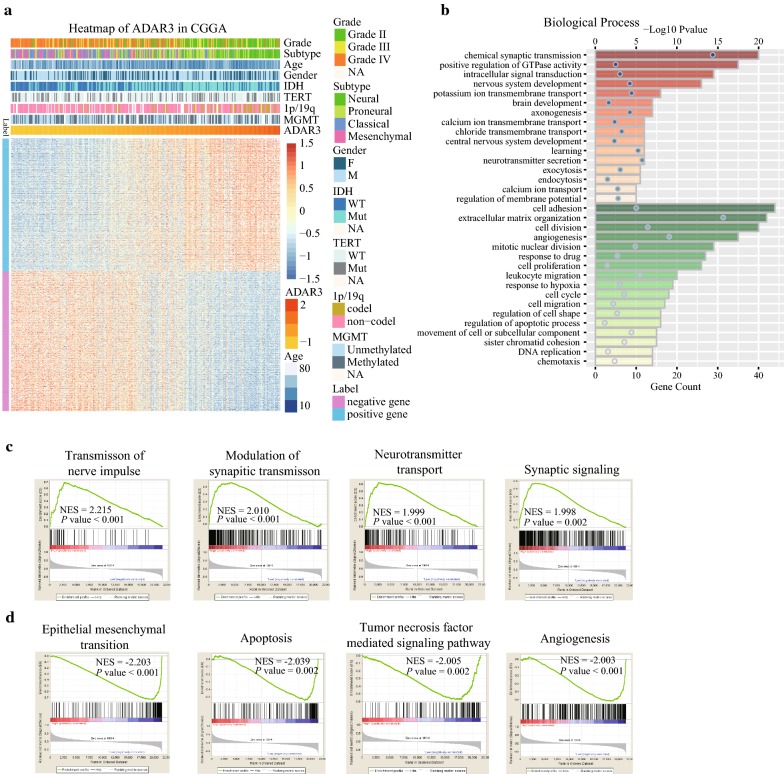
Functional annotation of ADAR3 in CGGA RNAseq data. **a** Heatmap of ADAR3 in CGGA RNAseq data. **b** Gene ontology analysis of the biological processes for ADAR3 by DAVID. Orange columns, ADAR3 positive related genes involved biological process; Green columns, ADAR3 negative related genes involved biological process. **c** GO terms enriched in high *ADAR3* expression group of glioma patients analyzed by GSEA. **d** GO terms enriched in low *ADAR3* expression group of glioma patients analyzed by GSEA. NES, normalized enrichment score

To illuminate the biological feature of glioma with different *ADAR3* expression, we performed Pearson correlation analysis to evaluate the correlation of *ADAR3* expression and other genes in CGGA sequencing dataset. Totally, the positively or negatively correlated genes with ADAR3 mRNA expression in CGGA RNAseq dataset were 844 and 891, respectively (|R| ≥ 0.5, Fig. 4a, Additional file 5: Table S1). The correlated genes were used for functional annotation analysis with DAVID and ranked by *P* value in increasing order. We found that ADAR3 positive related genes were mainly involved in normal biological process, such as chemical synaptic transmission, positive regulation of GTPase activity, intracellular signal transduction, nervous system development and several kinds of ion transport (Fig. 4b). While ADAR3 negative related genes were frequently involved in the processes of cell adhesion, cell division, extracellular matrix organization, angiogenesis, cell proliferation and response to drug (Fig. 4b). Then, GSEA was applied between the high- and low-expression level of ADAR3. Based on CGGA mRNA sequencing gene expression profile, we observed that GO terms including “transmission of nerve impulse”, “modulation of synaptic transmission”, “neurotransmitter transport” and “synaptic signaling” were significantly up-regulated in the high-expression group compared to the low-expression group (Fig. 4c). Moreover, “epithelial mesenchymal transition”, “apoptosis”, “tumor necrosis factor mediated signaling pathway” and “angiogenesis” were enriched in the low-expression group (Fig. 4d). These analyzes suggested that down-regulation of ADAR3 may potentiate the malignant transformation activity in glioma cells by affecting cell proliferation, angiogenesis or cell adhesion.

### *ADAR3* expression is positively correlated with A-to-I editing of GRIA2^Q607R^

**Fig. 5:**
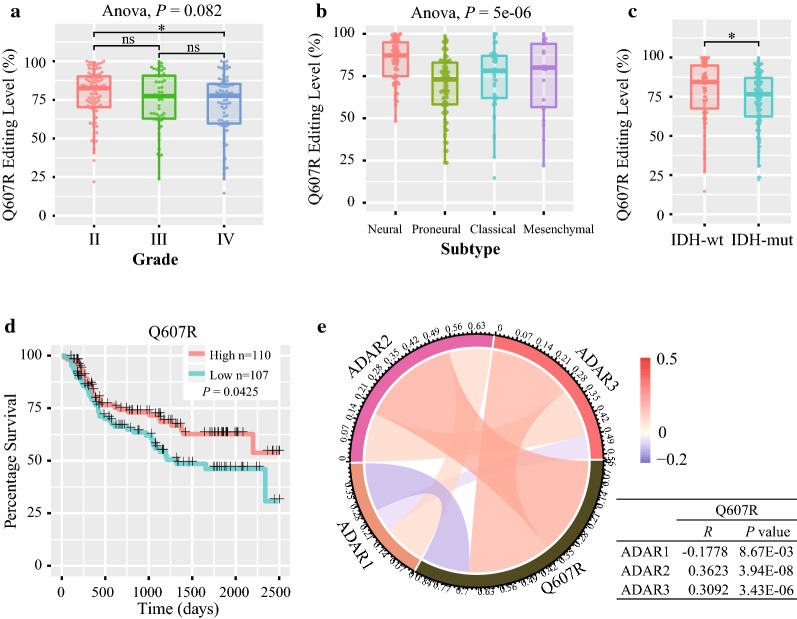
The feature of GRIA2^Q607R^ editing level in glioma samples. **a** The editing level of Q607R in different WHO glioma grade. **b** The editing level of Q607R in different TCGA subtype. **c** GRIA2^Q607R^ editing level in CGGA datasets according to IDH status. **d** Comparison of the OS between high and low GRIA2^Q607R^ editing group in patients with gliomas. **e** The relationship of ADAR1, ADAR2 and ADAR3 expression with GRIA2^Q607R^ editing level in glioma by Pearson analysis. OS, overall survival; *P < 0.05; ns, indicates no significant

The Q607R editing in the second transmembrane domain is nearly 100% throughout the mammalian brain, which is essential for normal receptor function [24]. Underediting of this site has been identified in malignant gliomas [25]. It is specifically edited by ADAR2, while ADAR3 directly competes with ADAR2 for binding to GRIA2 transcript, inhibiting RNA editing in U87 [14]. For analysis relationship between the Q607R editing level and ADAR family expression level in glioma samples, we first calculated the editing level of Q607R site in GRIA2 based on CGGA RNAseq database. Compared to Grade II gliomas, the editing level of GRIA2^Q607R^ is lower in Grade IV (*P *< 0.05, Fig. 5a). Meanwhile, the Q607R editing is related with different molecular subtype of glioma. Differential editing level at Q607R in GRIA2 has been identified in different TCGA subtypes (Anova, *P *= 5e-6, Fig. 5b). And the Q607R editing level is lower in IDH-mut glioma (*P *< 0.05, Fig. 5c). Moreover, the low Q607R editing level indicted a shorter overall survival time (Log-rank test, *P *= 0.0425, Fig. 5d). These results indicated that underediting of Q607R site in GRIA2 is a malignant marker for glioma based on a large cohort analysis.

Then, we calculated the relation of ADAR1, ADAR2 and ADAR3 expression with GRIA2^Q607R^ editing level in glioma by Pearson analysis. The ADAR2 and ADAR3 expression are positively correlated with the GRIA2^Q607R^ editing level (*R* = 0.3623, *P* = 3.94E−08; *R* = 0.3092, *P *= 3.43E−06; Fig. 5e), while ADAR1 expression is negatively correlated (*R* = − 0.1778, *P* = 8.67E−03, Fig. 5e). Collectively, these results indicated that the low expression of ADAR3 may induce unedited GRIA2 transcripts level which can promote cell migration and tumor invasion.

## Discussion

A-to-I editing is the most prevalent transcriptional modification in human cells, which is an endogenous process causing genetic diversity [5]. In vertebrates, ADAR1, ADAR2 and ADAR3 are main members that catalyzed A-to-I editing [26]. It has been demonstrated that ADAR enzymes are essential proteins by perturbing expression levels and functions in animal model. *ADAR1* deficient mice die during embryonic development, owing to defective hematopoiesis, widespread apoptosis, liver disintegration and an increasing activity of interferon signaling [27–29]. *ADAR2*^−*/*−^ mice became prone to seizures and died within 3 weeks after birth [30]. The catalytic activity of ADAR3 has not been demonstrated and *ADAR3*^−*/*−^ mice are viable and appear to be normal [5]. However, the catalytically inactive ADAR3, which localizes exclusively high in the brain, predominantly acts as an inhibitor of editing in the brain through competitive binds to dsRNA substrates [13, 14, 31].

Furthermore, a majority of transcripts encoding proteins involved in neurotransmission are often targets of A-to-I editing, resulting in changes in the amino acid sequence of protein and physiological function of these ion channels and receptors [11]. Currently, mutations or changes in expression induced disorder of ADAR activity have been linked to a variety of human diseases, ranging from neurological and neurodegenerative diseases, metabolic diseases, viral infections, autoimmune disorders to cancers [10]. Previously, ADAR2 has been reported to relate with glioblastoma in both children and adults. ADAR2 promotes *CDC14B* editing and overexpression in astrocytoma cells, leading to Skp2 degradation and upregulation of p21 and p27 proteins, consequently causing cells to accumulate in the G1 phase of the cell cycle [32]. ADAR2 deaminase activity is essential to inhibit glioblastoma proliferation and tumor growth [32]. Meanwhile, the impaired ADAR2 activity in GBM inhibits a subset of onco-miRNAs (miR221, miR222, miR-21, miR-376a and miR-589–3p) editing, leading to tumor cells proliferation, migration and invasion [33–35]. Recently, Oakes et al. have demonstrated that ADAR3 directly competed with ADAR2 for binding to *GRIA* transcript and inhibited RNA editing at the Q/R site of GRIA2 in glioblastoma [14], and this editing position of GRIA2 was substantially underedited in malignant human brain tumors compared with control tissues [25]. These suggested that ADAR3 may play a critical role in oncogenesis and development of glioblastoma. However, the clinical and molecular characterizations of ADAR3 in glioma still required further studies.

In this study, we analyzed the expression level of ADAR3 in four independent datasets including 1578 glioma patients. Most notably, *ADAR3* mRNA expression decreased along with WHO grade progression, suggesting that the expression of *ADAR3* gradually attenuated with the malignant increase of pathological glioma. Moreover, the *ADAR3* expression level was significantly highest in the phenotype of known favorable molecular, such as neural subtype and LGG IDH-mut and 1p/19q codeleted stratified patients. The association of ADAR3 with glioma progress indicates a tumor suppressor role of ADAR3 in the tumorigenesis and progression of glioma. However, an increase in ADAR3 protein expression in the tumor tissue compared to adjacent tissue was observed in 5 out of 6 glioblastoma patient samples in previous study [14]. Thus, inconsistencies between different studies suggest the possibility that ADAR3 protein expression or activity may be modulated by a post-transcriptional way. At the same time, further study will be needed to investigate ADAR3 protein expression based on a large cohort.

From the overall survival curve, the low expression of *ADAR3* indicated shorter overall survival time and lower survival rate in glioma patients. After divided the patients into LGG and GBM subgroup, the high expression of *ADAR3* is also a favorable indicator in LGG group, but not in GBM group. Furthermore, the high expression of ADAR3 was associated with longer overall survival time in LGG IDH-mut and 1p/19q non-codeleted and LGG IDH-wt patients. Combined with univariate and multivariate Cox analysis, *ADAR3* expression is an independent prognostic factor in patients with diffuse glioma, especially in LGG group. Recently, several genes have been reported to be prognostic factors in gliomas, such as IDH1 [36], FGFR3 [37], Notch1 [38]. Our research illuminated the clinical features of ADAR3 in diffuse glioma and confirmed that *ADAR3* expression had a guiding significance for the prognosis of patients with LGG.

Through an analysis of the biological process of ADAR3 in glioma, we found that ADAR3 play an important role in the normal biological process, such as signal transduction, chemical synaptic transmission, neurotransmitter transport and intracellular signal transduction. This is consistent with A-to-I editing targets, which are primarily transcripts of neurotransmitter receptors and ion channels proteins [11]. Meanwhile, downregulation of ADAR3 will promote cell proliferation, angiogenesis, cell adhesion and migration. This indicated that inhibition the expression of ADAR3 in brain cells would promote the cell malignant transition. Collectively, these data strongly support the tumor suppressor role of ADAR3 in glioma progression.

The reduced GRIA2 editing at Q607R site has been observed in malignant gliomas [25], and the unedited GRIA2 protein promotes cell migration and invasion in these cell lines [24]. Oakes et al. reported that overexpression of ADAR3 inhibited RNA editing at the Q607R site of GRIA2 in astrocyte and astrocytoma cell lines [14], which indicted the competitive inhibition of ADAR2 with ADAR3 on this site. Based on our clinical data, we found the editing level of GRIA2^Q607R^ is positively related with ADAR2 and ADAR3 mRNA expression, which is inconsistent with previous in vitro assay. These results indicated that the regulation of GRIA2 editing in gliomas is a more complex model than previous studies, and the tumor suppressor role of ADAR3 may partly related with the underedited level of Q607R.

## Conclusions

Our preliminary study demonstrates the clinical and molecular characterization of ADAR3 in glioma development and progression. Unlike other adenosine deaminases, ADAR3 is a favorable independent prognostic indicator of LGG patients. And it appeared to act as a tumor suppressor in glioma cells. Therefore, further studies are necessary to confirm its underlying molecular mechanisms in glioma.

## Additional files

**Additional file 1: Figure S1.** Expression analysis of ADAR1, ADAR2 and ADAR3 in diffuse gliomas in TCGA, GSE16011 and Rembrandt.

**Additional file 2: Figure S2.** ADAR3 expression in stratified patients in TCGA, GSE16011 and Rembrandt datasets.

**Additional file 3: Figure S3.** Survival analysis in stratified patients based on ADAR3 expression in TCGA, GSE16011 and Rembrandt datasets.

**Additional file 4: Figure S4.** Expression analysis of ADAR3 expression among CDKN2A status in LGG IDH-mut and 1p/19q non-codeleted patients based on TCGA dataset.

**Additional file 5: Table S1.** Genes that correlated with ADAR3 expression in CGGA RNAseq dataset.

## Abbreviations

LGG: lower-grade glioma
ADARs: adenosine deaminases acting on RNA
WHO: World Health Organization
A-to-I: adenosine to inosine
GBM: glioblastoma
CGGA: Chinese Glioma Genome Atlas
RNAseq: RNA sequencing
TCGA: The Cancer Genome Atlas
OS: survival time
DAVID: Database for Annotation, Visualization and Integrated Discovery
GSEA: gene set enrichment analysis
ROC: receiver operating characteristic
