# Supplementary figures and images for: ADAR3 expression is an independent prognostic factor in lower-grade diffuse gliomas and positively correlated with the editing level of GRIA2Q607R
Source: Cancer Cell Int. 2018 Dec 3;18:196. doi: 10.1186/s12935-018-0695-8 (PMC6276233; doi:10.1186/s12935-018-0695-8)

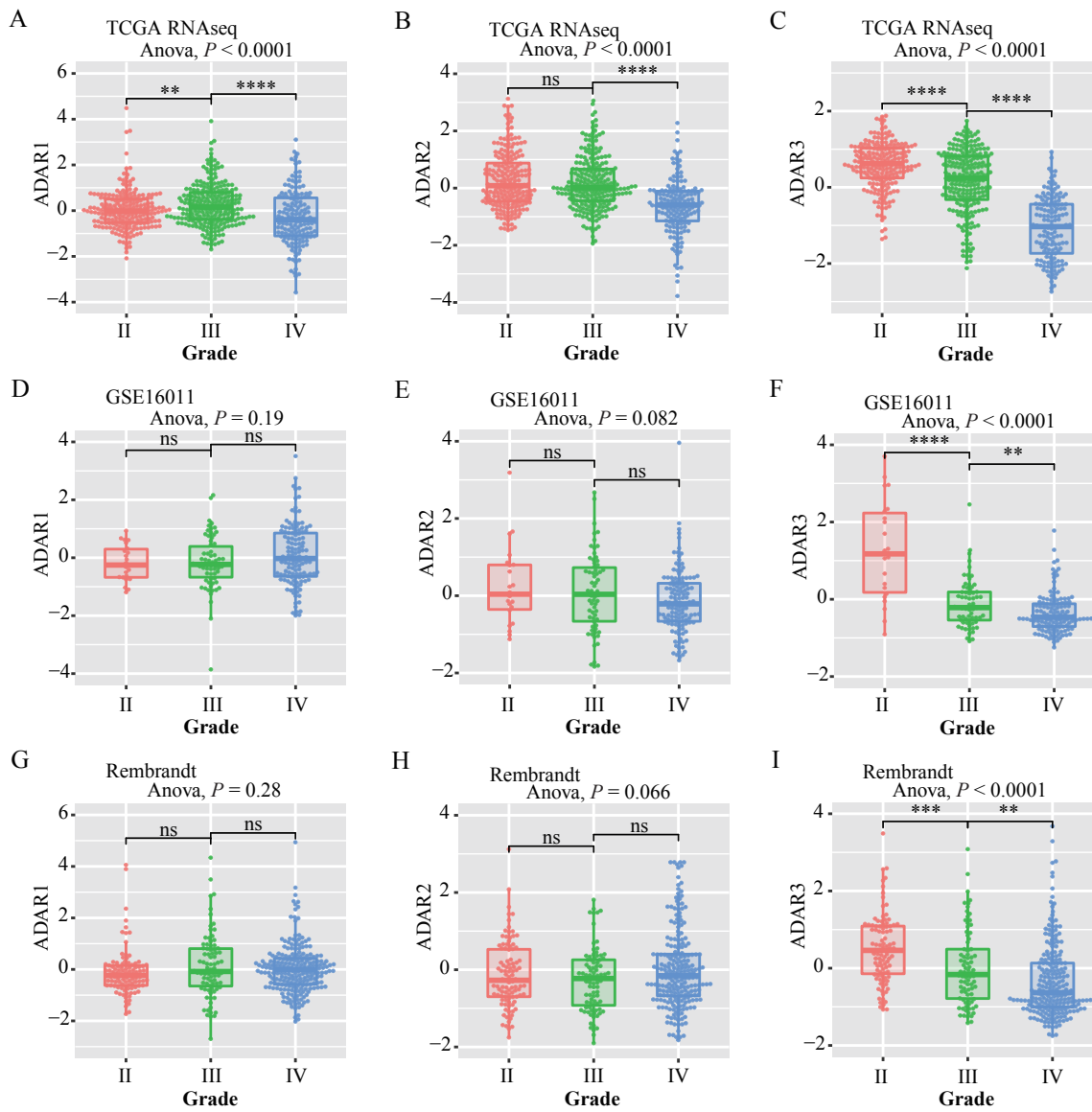

Supplement: Supplementary file 1 — Additional file 1: Figure S1. Expression analysis of ADAR1, ADAR2 and ADAR3 in diffuse gliomas in TCGA, GSE16011 and Rembrandt. [file 12935_2018_695_MOESM1_ESM.pdf]

A

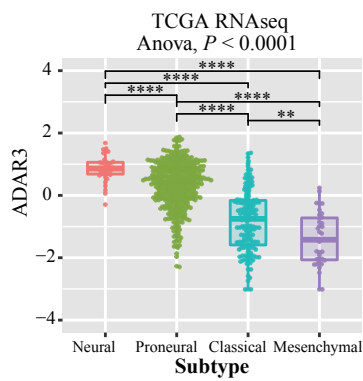

B

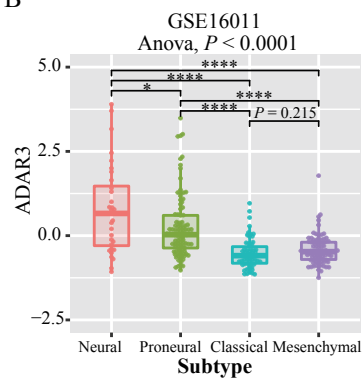

C

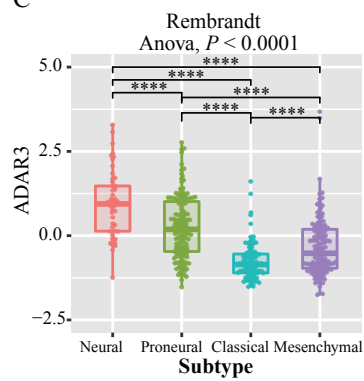

D

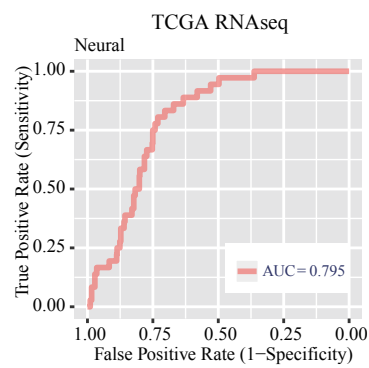

E

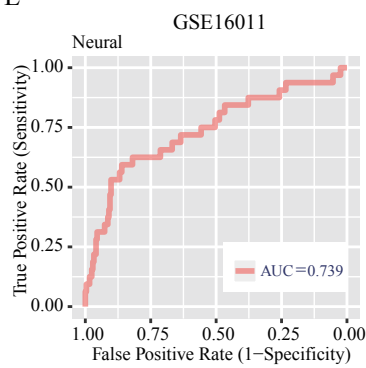

F

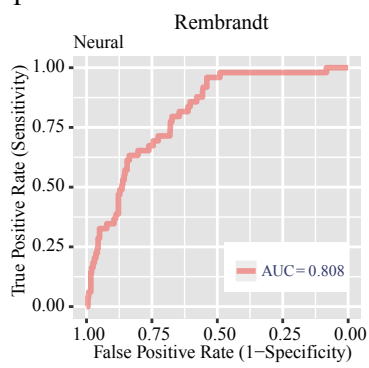

G

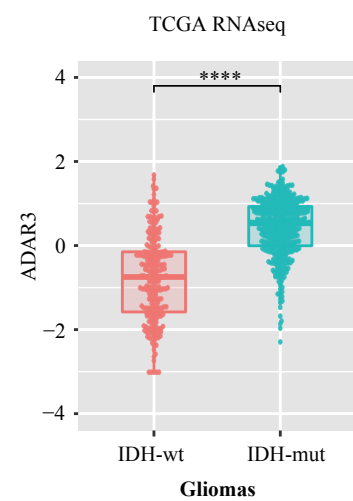

H

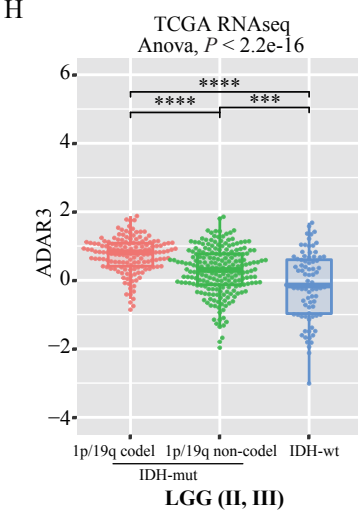

I

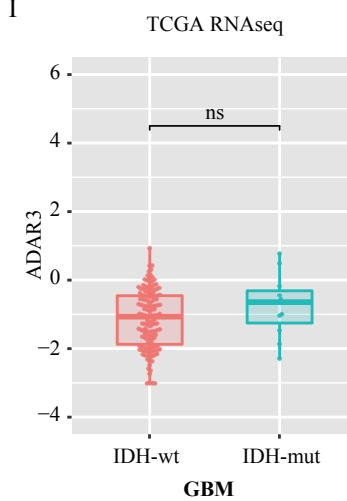

Supplement: Supplementary file 2 — Additional file 2: Figure S2. ADAR3 expression in stratified patients in TCGA, GSE16011 and Rembrandt datasets. [file 12935_2018_695_MOESM2_ESM.pdf]

A

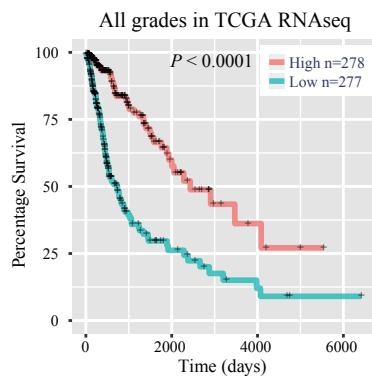

B

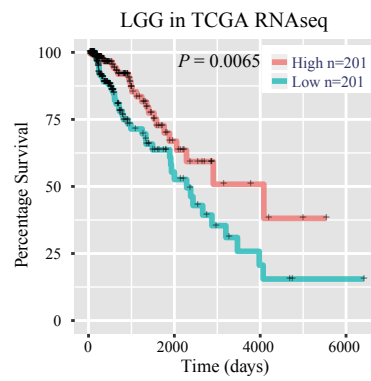

C

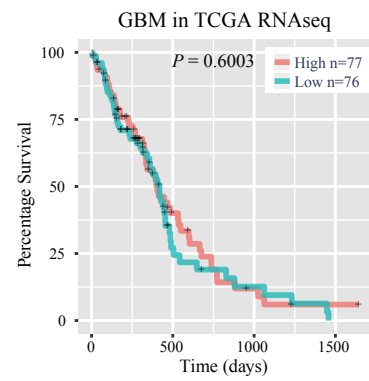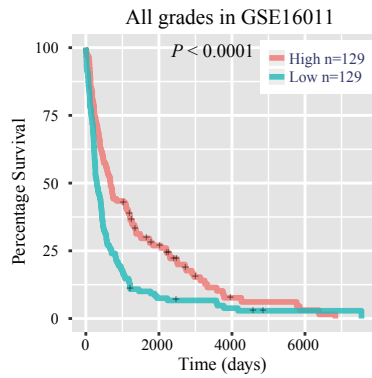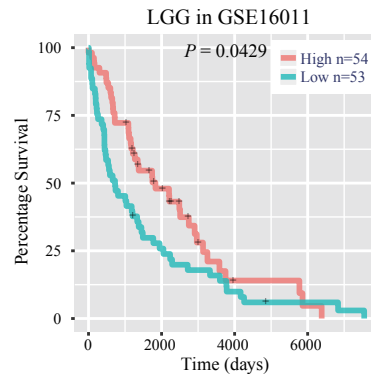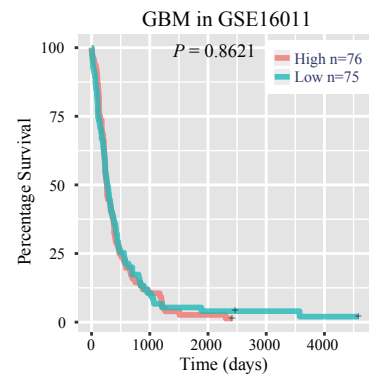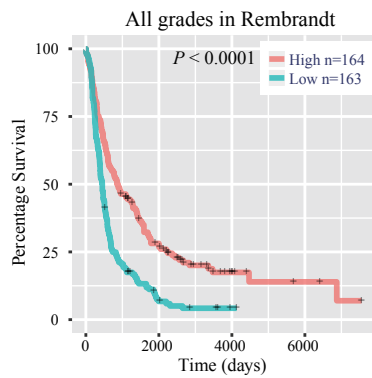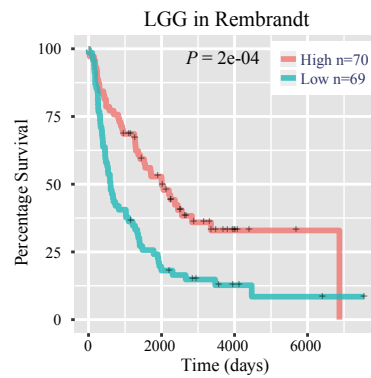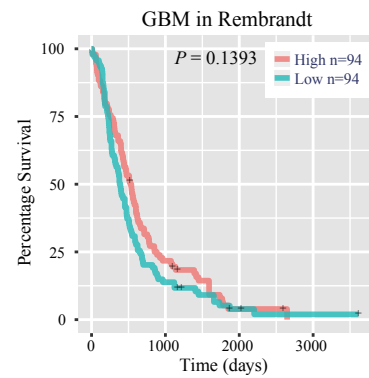

Supplement: Supplementary file 3 — Additional file 3: Figure S3. Survival analysis in stratified patients based on ADAR3 expression in TCGA, GSE16011 and Rembrandt datasets. [file 12935_2018_695_MOESM3_ESM.pdf]

A

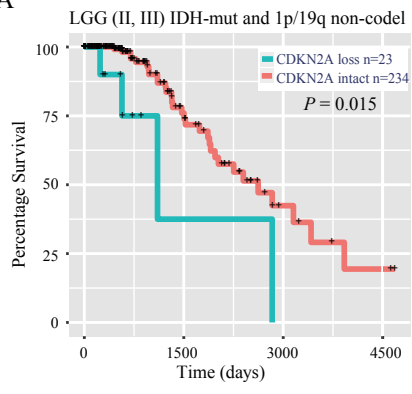

B

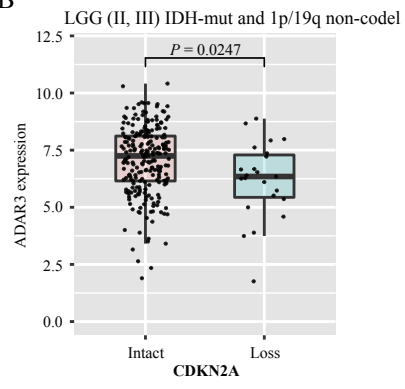

Supplement: Supplementary file 4 — Additional file 4: Figure S4. Expression analysis of ADAR3 expression among CDKN2A status in LGG IDH-mut and 1p/19q non-codeleted patients based on TCGA dataset. [file 12935_2018_695_MOESM4_ESM.pdf]
